# First case report of an intracortical lipoma in an adult tibia

**DOI:** 10.3892/ol.2013.1682

**Published:** 2013-11-12

**Authors:** SOO YONG KANG, HYOUNG-SEOK JUNG, JAE SUNG LEE, HO-JOONG JUNG

**Affiliations:** Department of Orthopedic Surgery, College of Medicine, Chung-Ang University, Seoul 140-757, Republic of Korea

**Keywords:** tibia, diaphysis, intracortical lipoma, magnetic resonance imaging

## Abstract

The current case report describes an adult male with an intracortical lipoma accompanied by cystic changes in the tibial diaphysis. To the best of our knowledge, intracortical lipoma in an adult tibia has not been previously described. An anteroposterior radiograph of the tibia revealed an osteolytic lesion on the diaphysis. Magnetic resonance imaging and computed tomography revealed that the lesion was located in the cortex and consisted of fat and cyst tissue. Surgical excision of the lesion confirmed diagnosis of an intracortical lipoma.

## Introduction

Intraosseous lipoma represents a relatively rare lesion composed of mature adipose tissue. The true incidence remains unknown, as the majority of lesions are asymptomatic and do not require medical attention. A number of authors have characterized these lesions as benign tumors of the medullary fat tissue (1,2). However, there have been four published cases of intracortical lipoma (3–6). To the best of our knowledge, intracortical lipoma in an adult tibia has not been previously described. The current case report describes an adult patient with symptomatic intracortical lipoma in the tibial diaphysis. The study was approved by the ethics committee of Chung-Ang University (Seoul, Korea). Written informed consent was obtained from the patient’s family.

## Case report

**Figure 1:**
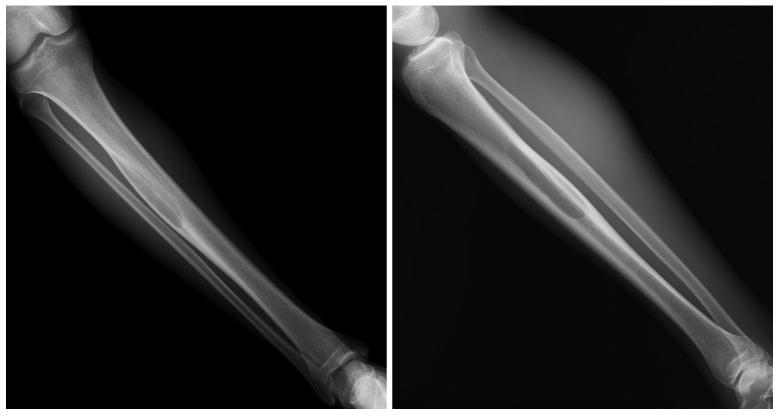
Anteroposterior and lateral radiographs showing a defined osteolytic lesion in the tibial diaphysis with mild bulging on the posterolateral side.

**Figure 2:**
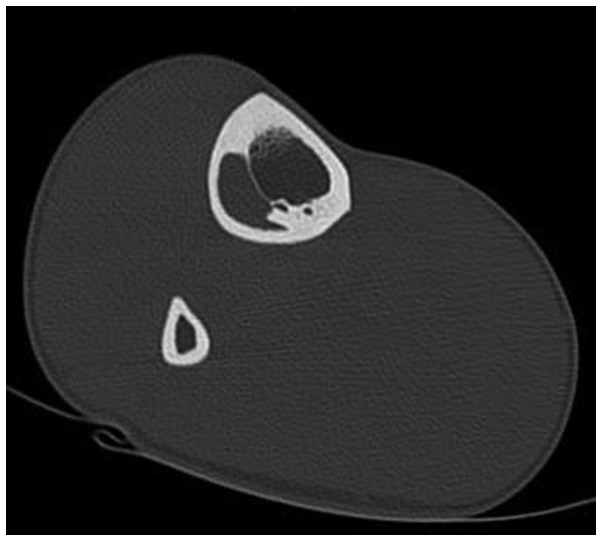
Axial CT image demonstrating an osteolytic lesion in the lateral tibial cortex which did not involve the medullary cavity. CT, computed tomography.

**Figure 3:**
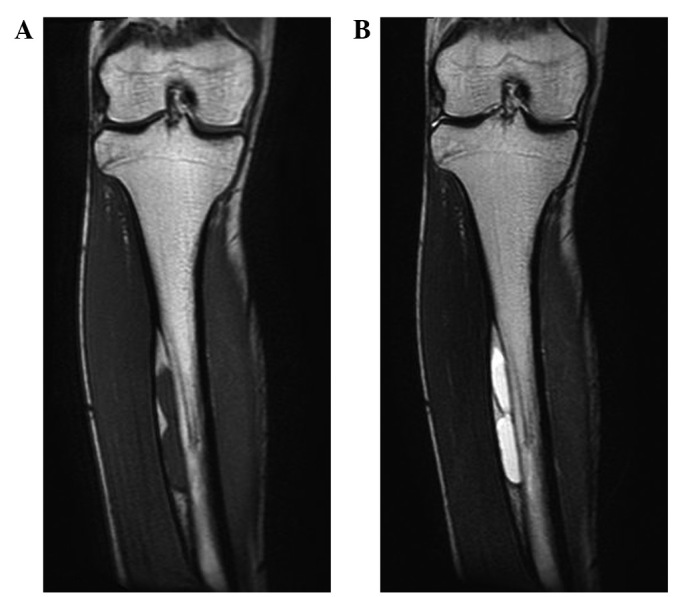
(A) Coronal T1-weighted MRI image revealing low-signal intensity in the central portion and high-signal intensity in the peripheral portion. (B) Coronal T2-weighted MRI image exhibiting high-signal intensity in the central and peripheral portions. MRI, magnetic resonance imaging.

A 23-year-old male presented to the Department of Orthopedic Surgery (College of Medicine, Chung-Ang University, Seoul) with pain and discomfort on the lateral aspect of the tibia. The patient denied history of trauma and had no past medical history. On physical examination, no visible or palpable mass, erythema, or warmth were noted. However, there was mild local tenderness on the lateral side of the tibia. Radiographs showed a well-defined osteolytic lesion in the tibial diaphysis with bulging on the posterolateral side. Plain radiographs did not show any evidence of periosteal reaction or cortical destruction (Fig. 1), however, confinement of the lesion to the cortex could not be confirmed. Computed-tomography (CT) scanning revealed a lucent lesion with regular margins, which did not involve the medullary cavity. The lesion was well-circumscribed and surrounded by sclerotic bone. The measurements were 90 mm in length and 20×10 mm in maximum diameter (Fig. 2). Magnetic resonance imaging (MRI) revealed that the lesion consisted of two different components, a central portion with low-signal intensity on T1-weighted MRI and high-signal intensity on T2-weighted MRI, and a peripheral portion with high-signal intensity on T1-weighted and T2-weighted MRI (Fig. 3). Technetium-99m scintigraphic scanning revealed increased isotope uptake at the lesion site. Based on the collective findings, the radiologist raised the possibility of an intracortical lipoma.

**Figure 4:**
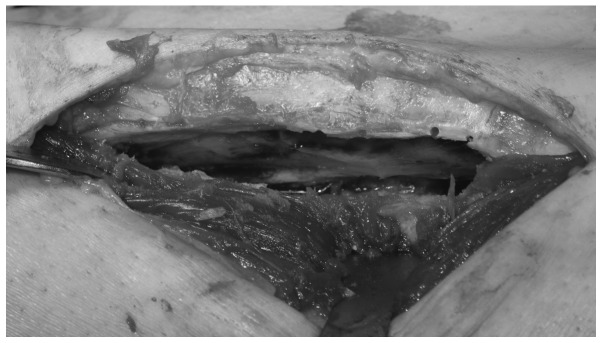
Intraoperative view of the intracortical lipoma. Following curettage, the true cortex was observed to be located beneath the lesion.

**Figure 5:**
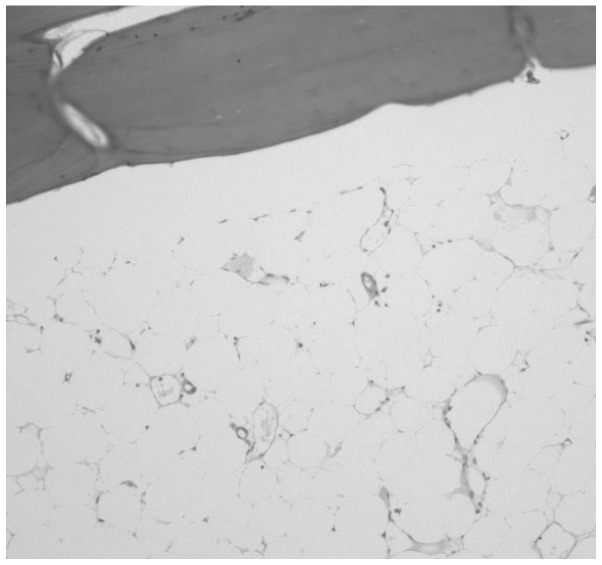
Histological appearance of an intracortical lipoma. Mature adipocytes and trabecular bone are shown (H&E stain; original magnification, ×200).

Excisional biopsy was performed to clarify the diagnosis. Following an incision on the anterolateral skin, periosteal dissection of the anterior compartment muscles was completed. The lesion was then identified to be bulging laterally and the surrounding cortical bone was removed. Macroscopically, the lesion showed cystic changes and was composed of fat tissue. Curettage was performed and the true cortex was located beneath the lesion (Fig. 4). Histopathological examination of the resected and curettage samples showed mature adipocytes with trabecular bone. No cellular atypia, fat necrosis or calcifications were observed (Fig. 5). Pain and discomfort disappeared during the postoperative period, and no tumor recurrence was observed at the 12-month postoperative follow-up.

## Discussion

Intraosseous lipoma is a rare, benign bone tumor that accounts for <0.1% of primary bone tumors (7). Differential diagnosis includes non-ossifying fibroma, simple bone cyst, fibrous dysplasia, enchondroma, aneurismal bone cyst, bone infarct and osteomyelitis (1,8–10). This particular tumor occurs most frequently in the lower limb, particularly in the calcaneus. Other common sites include long bone metaphyses (11). The majority of lesions are located intramedullary, and cortical bone involvement is rare. According to published reports, four cases of intracortical lipoma have been previously described. Of these cases, three patients were adults and all tumors were located in the femoral diaphysis (3,4,6). The remaining case report was of a seven-year-old pediatric patient with a tumor located in the tibial metaphysis (5). All four patients were female, two patients presented with pain and two lesions were found incidentally. The present case study appears to be the first report of an adult patient with an intracortical lipoma in the tibial diaphysis (Table I).

Radiographic appearance of intraosseus lipomas varies but can include well-defined osteolytic lesions with marginal sclerosis. Radionuclide bone scanning can be positive or negative and, therefore, has limited diagnostic utility (12). Although diagnosis of intracortical lipoma on plain radiographs remains challenging, CT and MRI are useful for detecting fat within the lesion and for helping to confirm a purely intracortical location (7). Lipoma fat can be easily recognized on MRI by high-signal intensity on T1- and T2-weighted images. Cyst signal intensity is low on T1-weighted MRI but high on T2-weighted MRI due to the fluid component. In the present patient, peripheral high-signal density on T1- and T2-weighted MRI scans was captured due to fat tissue. The central portion of the cystic change showed low-signal intensity on T1-weighted MRI and high-signal intensity on T2-weighted MRI. In operation filed, we could confirm that the radiological finding corresponded to the pathologic specimen.

Previously, Milgram (8) subdivided intraosseous lipomas into three stages. Stage 1 lesions are purely radiolucent and consist almost entirely of fat tissue. In stage 2 lesions, central calcifications with adjacent minor cystic degeneration in viable adipocytes are observed. Stage 3 lesions represent late cases in which fat has been devitalized with varying degrees of cyst formation, calcification and reactive bone formation. Although there were no calcific changes, we hypothesize that the histological observations of the current case are consistent with stage 3 lesions, due to the appearance of cystic changes.

The pathogenesis of intracortical lipomas remains controversial. A number of authors have suggested that lesions represent benign tumors of medullary adipose tissue (1,2). Others consider these lesions to be products of reactive changes secondary to infarct, infection or trauma (13). It has also been proposed that cyst formation results from degeneration of a pre-existing lipoma, or that a lipoma represents the late-stage of a simple bone cyst (11). However, none of the aforementioned theories adequately explain the underlying pathogenesis of an intracortical lipoma.

The majority of intraosseous lipomas can be managed conservatively. Surgery is indicated for symptomatic lesions, malignancy, or risk of pathological fracture. Surgical treatment usually consists of curettage and bone grafting (11). For the current case, surgical treatment was performed, as the lesion was large and the patient complained of mild pain. Furthermore, confirmation that the lesion was a benign, intracortical lipoma was not obtained.

In conclusion, the current report presents a case of an adult male with an intracortical lipoma accompanied by cystic changes in the tibial diaphysis. Intracortical lipoma represents a rare, benign bone tumor typically discovered incidentally. Although biopsy is required to confirm diagnosis, CT and MRI are useful imaging modalities to elucidate lesion characteristics and location. Intracortical lipomas should be included in the differential diagnosis of intracortical, osteolytic lesions of long bones.

## Figures and Tables

**Table I:** Summary of case reports of intracortical lipomas.

Study (year) [ref]	Age, years	Gender	Location	Radiological observations	Symptoms	Treatment
Downey *et al*(1983) [3]	34	Female	Diaphysis of femur	Incidental finding	Dense, separated lesion expanding the cortical outline of the diaphysis.	Surgical excision
Yamamoto *et al*(2002) [6]	74	Female	Diaphysis of femur	Thigh pain	Small osteolytic lesion in the lateral diaphyseal cortex.	Surgical excision
Lee *et al*(2007) [4]	31	Female	Diaphysis of femur	Incidental finding	Well-defined, expansile, radiolucent lesion with multiple septa in the diaphyseal cortex.	Surgical excision
Madhuri *et al*(2007) [5]	7	Female	Metaphysis of tibia	Proximal tibia pain	Sclerosis of the upper-anteromedial tibial cortex that contained a linear lytic lesion.	Surgical excision
Current study (2013)	23	Male	Diaphysis of tibia	Lateral tibia pain	Well-defined osteolytic lesion in the diaphysis of the tibia with mild bulging on the posterolateral side.	Surgical excision
